# P300: Blood splatters induced by using safety intravenous catheters

**DOI:** 10.1186/2047-2994-2-S1-P300

**Published:** 2013-06-20

**Authors:** T Horii

**Affiliations:** 1 Department of Infectious Diseases, Hamamatsu University School of Medicine, Hamamatsu, Japan

## Introduction

Various intravenous catheter (IC) products with safety mechanisms for prevention of needle stick injury are commercially available in Japan. However, risk of blood-borne infections to health care workers (HCWs) due to scattering blood when using these ICs has been pointed out.

## Objectives

We evaluated the level of scattering blood induced by using the safety ICs under a simulated condition to clarify the measures for prevention of blood-borne infections to HCWs.

## Methods

A mock arm was filled with bovine blood, adjusted to a blood pressure of 20 mmHg. A plastic seat (30 cm × 40 cm) was placed on the arm. An IC was inserted into the arm. Then, an inner needle was removed and/or the safety mechanism was activated. To collect splattered blood on the seat, it was divided into 12 square-shaped areas (10 cm × 10 cm) and then each area was wiped by using an adenosine triphosphate (ATP) swab test kit (LuciPac Pen, Kikkoman Biochemifa). ATP in the specimen was measured as fluorescent intensity, using a luminometer. Fluorescent intensity was converted to the amount of blood by using calibration curve. Student's t-test was used for statistical analysis of data.

## Results

Four out of 5 IC products induced blood splatter around the puncture site of the arm. Active safety ICs with retractable mechanisms of inner needles such as Insyte Autoguard BC (Bection Dickinson) and Supercath 5 (Medikit) induced blood splatter on the area ranging from 25 to 35 cm distance from the puncture site. Passive safety ICs with automatic mechanisms of covering needle tips after removing inner needles such as Introcan Safety 3 (B.Braun Melsungen) and Surshield SurFlash PS (Terumo) induced blood splatter when removing the inner needles by hands. The amount of splattered blood on the area ranging from 5 to 15 cm distance was significantly higher than that of the active-type products.

## Conclusion

Four IC products induced blood splatter when removing the inner needles and/or activating the safety mechanisms. Although splattered blood was a very small amount in microliter scale, these devices provided the risk of blood-borne infections to HCWs. To reduce the risk, standard precautions, including hand hygiene and use of gloves, should be complied whenever blood vessel puncture is carried out. Standardization of the procedure from puncture to connection with adapter of an infusion set is also required for each safety IC product.

## Disclosure of interest

None declared

